# The citizen preferences–positive externality trade-off: A survey study of COVID-19 vaccine deployment in Japan

**DOI:** 10.1016/j.ssmph.2022.101191

**Published:** 2022-08-17

**Authors:** Takashi Iida, Keisuke Kawata, Masaki Nakabayashi

**Affiliations:** Institute of Social Science, The University of Tokyo, Hongo 7–3–1, Bunkyo, 103–0033, Tokyo, Japan

**Keywords:** Herd immunity, Deservingness, Positive medical externality, Positive occupational externality, Geopolitical concerns, Immigrant discrimination

## Abstract

**Objective:**

Medicine is a scarce resource and a public good that benefits others by bettering patients’ health. COVID-19 vaccines in shortage are, 1) a scarce resource and 2) a public good with the positive externality of building herd immunity. These features are expected to drive citizens’ attitudes in opposite directions, exclusionist and inclusionist, respectively. Scarcity would drive citizens’ exclusionism, while the positive externality might mitigate exclusionism.

**Setting and design:**

We recruited 15,000 Japanese adults and asked them to rank, in the context of a COVID-19 vaccine shortage, the deservingness of hypothetical vaccine recipients who differed according to 1) citizenship status, 2) visa type and duration of stay (if foreign), 3) occupation, 4) age, 5) whether they lived with a child, and 6) whether they lived with an elderly individual. Citizenship options were Japanese, Chinese, Taiwanese, South Korean, American, or European. The occupations were healthcare, education, other employed, self-employed, or not employed. The 6 attributes were randomly combined, and respondents were shown 3 hypothetical vaccine recipients: one was Japanese, and the others were foreigners.

**Treatments:**

First, through a conjoint design, we created hypothetical vaccine recipients whose attributes were randomized except for the benchmark citizenship, Japanese national. Second, we randomly presented two scenarios for vaccination payments: 1) billed at cost or 2) fully subsidized by the government.

**Results:**

1) Whether the vaccines were billed at cost or fully subsidized did not affect the rankings of deservingness. 2) Japanese citizenship was prioritized. 3) The penalty for being a foreigner was higher for individuals from nations with which Japan has geopolitical tensions. 4) Working in health or education reduced the penalty on foreigners, indicating that the positive externality related to occupation amplifies the positive externality associated with vaccination and mitigates exclusionist attitudes.

**Conclusions:**

The positive occupational externalities that amplify the positive externality of vaccination substantially allay the foreigner penalty.

## 1 Introduction

Medicine in general has two sides. First, it is a public good due to its positive externality on others’ health. This is especially the case when building herd immunity against infectious diseases. Furthermore, positive medical externalities might be multiplied by the positive social externalities of treatment recipients. This would justify the prioritization of vaccination for healthcare and educational workers because their positive occupational externality arising from their contact with patients and students multiplies the positive medical externalities of vaccination. Second, however, medicine tends to be a highly scarce resource due to its cost.

The institutional variations in healthcare insurance observed between advanced nations reflect differing emphases on these factors. Japan’s National Health Insurance Act of 1958, an extension of the National Health Insurance Act of 1938, is a universal, compulsory health insurance program that covers all residents in Japan, regardless of their citizenship and regulates the entire medical market. Any person who lives in Japan is entitled to the same care at the same price, which is regulated by the government. Thus, the Japanese system considers medicine a public good and that keeping foreigners and citizens equally healthy is in the nation’s best interest. At the other extreme is the US, where the market mechanism is left to allocate the vast range of medical services because it is considered the best system for allocating scarce resources.

However, whether ordinary Japanese people feel that medicine is a public good is a separate question. Indeed, the COVID-19 pandemic has revealed the global vulnerability of constitutional and inclusive institutions through violations of apparently established institutional norms (Edgell et al., 2021). A sensitive issue involves conflicts between the values of constitutional democracies and the security measures implemented against COVID-19, which is referred to as the security–liberty trade-off (Koyama, 2021; Pennington, 2021; Pereira & Stornelli, 2022; van Vark, 2021), a classical issue dating back to Hayek (2007) (Hayek, 2007, p. 155). The issue of whether to allow the tracking of SMS/phone communication to detect infection routes is particularly serious and has created divides both between and within advanced democracies (Beduschi, 2021; Fahey & Hino, 2020; Ghose et al., 2022; Kawata & Nakabayashi, 2022; Tran & Nguyen, 2021; Zhang et al., 2020).

We argue that another sensitive issue is the balance between considerations of scarcity and the positive externality of medicine (vaccination, in this case). To protect the citizens of a constitutional democracy, its government must obtain vaccines if domestic production capacity is not adequate and may even need to compete with other nations. At the same time, once a nation obtains vaccines, that nation should vaccinate citizens and immigrants equally depending on the strength of the positive externality associated with vaccinating an individual through the recipient’s contacts with others if the shortage of vaccines is severe. Because of the positive externality of building herd immunity, exhibiting openness and inclusiveness within a nation greatly enhances that nation’s safety. A country that is inclusive toward a broader range of people is a safer nation for every citizen and every guest.

During the early stages of the pandemic, addressing vaccine hesitancy was a serious issue (Baccolini et al., 2021; Falcone et al., 2022; Hara et al., 2021; Kawata & Nakabayashi, 2021; Kreps et al., 2020; Latkin et al., 2021; Motta, 2021; Niño et al., 2021; Niu et al., 2022; Schwarzinger et al., 2021; Stöckli et al., 2022). A shared concern in the work on vaccine hesitancy is that such reluctance might mitigate the positive externality associated with vaccination. In addition, the equitable allocation of scarce resources as related to vaccine distribution and ICU triage practices that considered age, occupation, ethnicity, and citizenship was an issue (Knotz et al., 2021b; Larsen & Schaeffer, 2021; Reeskens et al., 2021; Vinay et al., 2021).

Once vaccines for COVID-19 were rolled out, the fair prioritization of vaccination (Buckner et al., 2021; Duch et al., 2021; Persad et al., 2020) and equitable distribution (Emanuel et al., 2020; Zard et al., 2021) were called for. Although the argument for the equitable distribution of vaccines was primarily made in a global context and focused on the need to distribute vaccines to developing nations to build global herd immunity, the same issue remained a challenge within nations. To make each nation safer, an inclusive vaccination regime must be implemented.

A common backdrop for the issues discussed above is the concern that national interests might dominate in issues related to international relations and that within a nation, the interests of the majority (that is, prime-aged citizens) might overwhelm those of minorities such as immigrants or elderly people. Findings from previous works largely validate these concerns.

Works on the allocation of COVID-19 medical treatments belong to a subset of studies on deservingness and the rationing of welfare programs and medical treatments (Knotz et al., 2021a; Larsen & Schaeffer, 2021; Reeskens & van der Meer, 2019; van Oorschot, 2000), which includes the distribution of organs for transplant (Bramstedt, 2006; Childress, 2001; Gutmann & Land, 1997; O’Dell et al., 2019; Wall et al., 2020). As found in studies on the COVID-19 pandemic, people tend to assign greater deservingness to people with greater proximity to themselves, notably fellow citizens.

Therefore, a critical issue is whether and to what extent the positive externalities of medicine, particularly vaccination, are understood by the citizens of each nation. In reality, medical resources are scarce despite their status as public goods. When welfare is considered to be a type of redistribution of scarce resources, citizens’ perceptions of deservingness tend to be distorted toward people similar to themselves (Knotz et al., 2021a). If ICU beds are considered a scarce resource, it is not surprising that competition for ICU beds leads to attitudes of exclusion toward foreigners (Knotz et al., 2021b). However, as rational creatures, we should also be able to understand the positive externalities of medicine, and this understanding should lead us toward an attitude of inclusion that benefits both guests and citizens, which makes our nation safer. Citizens’ priorities might involve a trade-off between positive externalities and the perceived higher deservingness of fellow citizens.

Previous works on vaccine development have found that prepandemic geopolitical concerns cast a shadow. Kreps et al. (2020), Motta (2021), Schwarzinger et al. (2021), Kawata and Nakabayashi (2021), Vanhuysse et al. (2021), and Stöckli et al. (2022) among others, found that people in advanced democracies such as the US, Japan, Germany, France, and Sweden tended to avoid proposed COVID-19 vaccines developed in nations with which they had geopolitical concerns, such as China and Russia. If this attitude originated from the quality of vaccine licensing or medical science in general in Russia and China, it could be justified as a rational judgment. However, once effective and safe vaccines became available, Chinese recipients in those advanced democracies should have been welcomed by rational citizens in order to benefit both Chinese guests and fellow citizens. We also test this argument.

## 2 Methods

### 2.1 Randomized conjoint analysis

Our hypothetical candidates for vaccine receipt were generated by a randomized conjoint experimental design (Hainmueller et al., 2014). Respondents were shown three hypothetical vaccine recipients as described in section 2.4.1 below and were requested to choose their priority for vaccination, ranking them from first to third. The third-ranked recipient was given the lowest priority. One of the three candidates was always Japanese, and the other two were Chinese, Taiwanese, South Korean, American, or EU citizens. In this sense, our design was a partially randomized conjoint experiment. Other attribute levels such as occupation and age were randomly assigned. Each respondent completed five rounds of the ordering task.

### 2.2 Survey

The survey consisted of two parts. Respondents were requested to rank the three hypothetical vaccine recipients generated by our partially randomized conjoint design over five rounds as described above. Then, they were asked about their demographic, political, and socioeconomic characteristics as well as whether they had been vaccinated against COVID-19.

### 2.3 Aims

As in the previous works discussed, we expected that our respondents would perceive fellow citizens to be more deserving of receiving an allocated medical treatment presented as a scarce resource. Since the three candidates shown to the respondents always included one Japanese individual, our conjoint design measured how much Japanese respondents penalized foreigners.

Although Japan is one of the most frequent destinations for immigration among OECD countries (Chiavacci, 2016, pp. 233–249), the Japanese government does not use the word “immigrants” (Roberts, 2017). Instead, those who migrate to Japan are described as foreigners with a long-term stay working visa or a highly skilled professional visa or as “permanent residents”. Thus, throughout this paper, foreigners with working visas or highly skilled professional visas are equivalent to “immigrants” in other advanced nations. Therefore, if there is a deservingness penalty for foreigners with working visas or highly skilled professional visas, it is equivalent to the penalty on immigrants in other advanced nations. Furthermore, previous works have demonstrated that Japanese citizens exhibit attitudes of exclusion toward and discrimination against immigrants, as do the citizens of other advanced nations (Holbrow & Nagayoshi, 2018; Igarashi & Nagayoshi, 2022). How to tame these exclusion-oriented sentiments within citizens’ priorities is now an issue in Japan. Our design investigates whether and how we could benefit from the positive externality of building herd immunity against COVID-19 by taming exclusionist attitudes.

We have two goals for our treatments. First, we intend to identify whether the positive externalities arising from foreigners’ occupations, which could amplify the positive externalities of vaccination, could mitigate the foreigner penalty. Second, we attempt to identify whether vaccination billing at cost or government subsidization affects the magnitude of the foreigner penalty.

### 2.4 Treatments

#### 2.4.1 Treatment 1: attributes of the conjoint experiment

In our conjoint experiment, we asked the following question to all respondents:

In cases in which there is a shortage of vaccines against COVID-19 due to the spread of variants of the virus, which people do you think should be prioritized for vaccination? Please rank the three candidate recipients described below. If you cannot rank them, please indicate the same rank.

The question defines a situation in which COVID-19 vaccines remain a scarce resource due to SARS-CoV-2 variants, such as cases in which updates to existing vaccines or booster shots become necessary. This question was followed by a sentence regarding the cost of vaccination billed at cost or fully subsidized by the government, as described in 2.4.2.

Below the question, three candidate recipients were shown to each respondent in each of the five rounds. Each candidate exhibited six attributes that had two to six possible levels, as described in Table 1. The levels were randomly chosen. One of the three candidates was always a Japanese citizen, although the other attributes were randomly chosen. With this treatment, we intended to identify whether the foreigner penalty was mitigated by attributes other than citizenship, such as occupation, age, visa type, and family composition and whether the foreigner penalty differed by whether Japan and the nation of origin had geopolitical tensions. Kobayashi et al. (2014) found that Japanese attitudes toward the deservingness of foreigners for naturalization depend on the applicants’ socioeconomic status. We capture socioeconomic status with visa types; a “highly skilled” visa corresponds to a higher socioeconomic status.

**Table 1:** Conjoint design: Attributes of hypothetical COVID-19 recipients.

Attribute	Level					
	1	2	3	4	5	6
Occupation	Healthcare	Education/childcare	Employed	Self-employed	Not employed	
Citizenship	Japan	United States of America	People’s Republic of China (China)	Republic of China (Taiwan)	Republic of Korea (South Korea)	European Union
Residency status	Japanese citizen	Short-term stay visa (tourism, business, etc.)	Highly skilled professional visa	Working visa (education, research, medical, nursing, intracompany transfer, etc.)	Permanent resident	
Age	17–30	31–45	46–64	65 or over		
Lives with a child aged 5 or under	Yes	No				
Lives with an elderly individual aged 65 or over	Yes	No				

For occupation, we expected healthcare and education/childcare occupations to have larger externalities due to those workers’ contacts with patients, students, and children. Age and family composition captured the infection risk of the respondents themselves and their families.

Regarding the geopolitical context, while the US is Japan’s only formal ally, the Japanese navy has conducted joint drills with the British, Australian, French, and German navies as well as the US navy.^1^ While deepening its socioeconomic relationships with China, Japan, along with the US, has strengthened its political support for Taiwan’s status as an autonomous democracy (Noble, 2005). For China, the Japanese archipelago is located at the gateway to the Western Pacific, resulting in increased tension between China and the US–Japan alliance (Erickson & Wuthnow, 2016; Fanell, 2019), adding to territorial disputes (Pajon, 2017). Although South Korea is an ally of the US, its citizens have anti-Japanese sentiments due to Japan’s annexation of Korea from 1910 to 1945; these feelings are embedded in and enhanced by domestic political dynamism (You & Kim, 2020), and Japanese citizens recognize this. In summary, Japan maintained geopolitically good prepandemic relationships with the US, Europe, and Taiwan but not necessarily with China or South Korea. Note that the socioeconomic interdependence between China and Japan has deepened, and Japan has accommodated the socioeconomic rise of China (Jerdén & Hagström, 2012). Japan and Taiwan share democratic values with South Korea. Furthermore, Chinese, Taiwanese, Korean, and Japanese citizens share an East Asian culture and belong to the same race. Thus, the perceived prepandemic tensions in East Asia were predominantly geopolitical (Gong & Nagayoshi, 2019).

#### 2.4.2 Treatment 2: two scenarios

We randomly chose and showed one of the two scenarios described below to the respondents, with a probability of 0.5 for each scenario in each round. We asked the respondents to rank the deservingness of the hypothetical vaccine recipients.

**Scenario 1** In cases in which there is a shortage of vaccines against COVID-19 due to the spread of variants of the virus, which people do you think should be prioritized for vaccination? Please rank the three candidate recipients described below. If you cannot rank them, please indicate the same rank. Vaccination is billed at cost.

**Scenario 2** In cases in which there is a shortage of vaccines against COVID-19 due to the spread of variants of the virus, which people do you think should be prioritized for vaccination? Please rank the three candidate recipients described below. If you cannot rank them, please indicate the same rank. Vaccination costs are paid in full by the government.

The difference between the two scenarios lies only in the last sentence: whether vaccination is billed at cost or entirely subsidized by the government. We adopted this treatment to identify whether the prioritization of fellow citizens over foreigners indicated by previous works depends on how the medical treatments are financed.

### 2.5 Data collection

We recruited a nonprobability sample of 15,000 Japanese adults through a survey company, Rakuten Insight, Ltd.^2^ We conducted the survey from November 8 to 24, 2021. The median response time per respondent was 11 min and 28 s.

### 2.6 Descriptive statistics

Table 2 shows the demographic characteristics of our respondents and their experience with vaccination against COVID-19.

**Table 2:** Descriptive statistics for background characteristics: Demographics.

Statistic	N	Mean	St. Dev.	Min	Max
Age	15,000	47.954	13.795	18	79
Gender (1 if female, 0 otherwise)	15,000	0.492			
Unmarried (1 if yes, 0 otherwise)	14,977	0.292			
Married (1 if yes, 0 otherwise)	14,977	0.622			
Divorced or bereaved (1 if yes, 0 otherwise)	14,977	0.086			
Number of children	14,956	1.092	1.126	0	5
Number of older siblings	8,870	0.712	1.001	0	12
Number of younger siblings	8,864	0.749	0.879	0	10
Vaccinated against COVID-19 (None: 0, first dose: 1, second dose: 2)	14,973	1.725	0.670	0	2

Note that the maximum number of children reportable in our questionnaire was “5 or more”, so “5” might include more than 5 children.

Table 3 presents the descriptive statistics on the working status of our respondents.

**Table 3:** Descriptive statistics for background characteristics: Working status.

Statistic	N	Mean
Working status (1 if at work, 0 otherwise)	14,922	0.738
Regular worker (1 if regular worker, 0 otherwise)	11,000	0.605
Board member (1 if board member, 0 otherwise)	11,000	0.021
Self-employed (1 if self-employed, 0 otherwise)	11,000	0.088
Worker: Non regular (1 if non regular worker, 0 otherwise)	11,000	0.286
Employee: No title (1 if no title, 0 otherwise)	9,585	0.674
Employee: Leader (1 if group leader, 0 otherwise)	9,585	0.043
Employee: Assistant manager (1 if assistant manager, 0 otherwise)	9,585	0.089
Employee: Department chief (1 if department chief, 0 otherwise)	9,585	0.083
Employee: Division manager (1 if division manager, 0 otherwise)	9,585	0.055
Size of employer: 1–4 employees (1 if yes, 0 otherwise)	9,598	0.039
Size of employer: 5–29 employees (1 if yes, 0 otherwise)	9,598	0.152
Size of employer: 30–99 employees (1 if yes, 0 otherwise)	9,598	0.157
Size of employer: 100–499 employees (1 if yes, 0 otherwise)	9,598	0.209
Size of employer: 500 or over (1 if yes, 0 otherwise)	9,598	0.374
Employer: Government (1 if yes, 0 otherwise)	9,598	0.069

Table 4 presents the descriptive statistics for our respondents’ political positions.

**Table 4:** Descriptive statistics for background characteristics: Political position.

Statistic	N	Mean	St. Dev.	Min	Max
Support Liberal Democratic Party (1 if yes, 0 otherwise)	14,984	0.233			
Support Constitutional Democratic Party (1 if yes, 0 otherwise)	14,984	0.064			
Support National Democratic Party (1 if yes, 0 otherwise)	14,984	0.021			
Support Clean Government Party (1 if yes, 0 otherwise)	14,984	0.023			
Support Party for Restoration (1 if yes, 0 otherwise)	14,984	0.109			
Support Japanese Communist Party (1 if yes, 0 otherwise)	14,984	0.028			
Independent (1 if yes, 0 otherwise)	14,984	0.486			
Degree of dissatisfaction with current politics (5: most dissatisfied to 1: satisfied)	14,973	3.784	1.036	1	5
Individual interest vs. public interest (4: strongly individual to 1: strongly public and 0 neither)	14,956	1.966	1.168	0	4
Support for welfare state (4: largest government possible to 1: smallest government possible)	14,864	2.569	1.355	0	4
Self-perceived degree to which political views lean right (10: right to 0: left)	14,612	5.207	1.481	0	10

Table 5 presents the highest degree earned and the self-perceived social status of our respondents.

**Table 5:** Descriptive statistics for background characteristics: Highest degree earned and self-perceived social status.

Statistic	N	Mean	St. Dev.	Min	Max
Junior high school (1 if yes, 0 otherwise)	14,978	0.014			
High school (1 if yes, 0 otherwise)	14,978	0.228			
Some college (1 if yes, 0 otherwise)	14,978	0.124			
2-year college (1 if yes, 0 otherwise)	14,978	0.089			
Technical 2-year college (1 if yes, 0 otherwise)	14,978	0.012			
4-year college (1 if yes, 0 otherwise)	14,978	0.468			
Graduate school (1 if yes, 0 otherwise)	14,978	0.064			
Self-perceived social status (highest: 0 to lowest: 10)	14,864	2.245	1.134	0	4

Table 6 shows descriptive statistics for our respondents’ income.

**Table 6:** Descriptive statistics for background characteristics: Income.

Statistic	N	Mean
Income: Less than 0.5 million yen (1 if yes, 0 otherwise)	14,964	0.158
Income: 0.5–0.99 million yen (1 if yes, 0 otherwise)	14,964	0.075
Income: 1–1.49 million yen (1 if yes, 0 otherwise)	14,964	0.073
Income: 1.5–1.99 million yen (1 if yes, 0 otherwise)	14,964	0.054
Income: 2–2.49 million yen (1 if yes, 0 otherwise)	14,964	0.082
Income: 2.5–2.99 million yen (1 if yes, 0 otherwise)	14,964	0.064
Income: 3–3.99 million yen (1 if yes, 0 otherwise)	14,964	0.122
Income: 4–4.99 million yen (1 if yes, 0 otherwise)	14,964	0.107
Income: 5 million yen or over (1 if yes, 0 otherwise)	14,964	0.265
Household income: Less than 0.5 million yen (1 if yes, 0 otherwise)	14,987	0.031
Household income: 0.5–0.99 million yen (1 if yes, 0 otherwise	14,987	0.012
Household income: 1–1.49 million yen (1 if yes, 0 otherwise)	14,987	0.024
Household income: 1.5–1.99 million yen (1 if yes, 0 otherwise)	14,987	0.030
Household income: 2–2.49 million yen (1 if yes, 0 otherwise)	14,987	0.053
Household income: 2.5–2.99 million yen (1 if yes, 0 otherwise)	14,987	0.047
Household income: 3–3.99 million yen (1 if yes, 0 otherwise)	14,987	0.111
Household income: 4–4.99 million yen (1 if yes, 0 otherwise	14,987	0.121
Household income: 5–5.99 million yen (1 if yes, 0 otherwise)	14,987	0.117
Household income: 6–6.99 million yen (1 if yes, 0 otherwise)	14,987	0.091
Household income: 7–7.99 million yen (1 if yes, 0 otherwise)	14,987	0.090
Household income: 8–8.99 million yen (1 if yes, 0 otherwise)	14,987	0.068
Household income: 9–9.99 million yen (1 if yes, 0 otherwise)	14,987	0.055
Household income: 10 million yen or over (1 if yes, 0 otherwise)	14,987	0.150

For comparison, Table A6 presents a summary of demographic characteristics surveyed by the 2020 population census, administered by the Ministry of Internal Affairs and Communications of the Government of Japan. Table A7 gives the household income distribution for 10,000 respondents to the National Livelihood Survey 2018 administered by the Ministry of Health, Labour and Welfare of the Government of Japan. Our sample has a slightly denser distribution near the high end of the range.

## 3 Estimation strategy

### 3.1 Effects of changes in attributes

Let ${Α}_{j}$ denote a hypothetical vaccine recipient with the six attributes described in Table 1, and let ${Α}_{j^{\prime }}$ and ${Α}_{j^{\prime \prime }}$ denote alternative hypothetical recipients. The citizenship status of one of ${Α}_{j}$, ${Α}_{j^{\prime }}$, and $A_{j^{\prime \prime }}$ is “Japanese citizen”.

Let us consider the ranking of the recipient by respondent *i* in round *r*, $Y_{i,j,r}^{\mathrm{order}}\left(A_{j}^{i,r}\right)$, where $A_{j}^{i,r}$ denotes a hypothetical recipient shown to respondent *i* in round *r*, *r* ∈ [1, 5]. If respondent *i* in round *r* prioritizes $A_{j}^{i,r}$ over $A_{j^{\prime }}^{i,r}$ and prioritizes $A_{j^{\prime }}^{i,r}$ over $A_{j^{\prime \prime }}^{i,r}$ such that

$$A_{j}^{i,r}{≻}_{i}A_{j^{\prime }}^{i,r}{≻}_{i}A_{j^{\prime \prime }}^{i,r},$$

then

(1) $$\left\{\begin{array}{cc} & Y_{i,j,r}^{\mathrm{order}}\left(A_{j}^{i,r}\right)=1\\ & Y_{i,j,r}^{\mathrm{order}}\left(A_{j^{\prime }}^{i,r}\right)=2\\ & Y_{i,j,r}^{\mathrm{order}}\left(A_{j^{\prime \prime }}^{i,r}\right)=3. \end{array}\right)$$

We review the respondents’ prioritization of recipients by estimating the average order for each hypothetical recipient such that

(2) $$\tau^{\mathrm{order}}\left(A_{j}^{i,r}\right)=E\left[Y_{j}^{\mathrm{order}}\left(A_{j}^{i,r}\right)\right].$$

We estimate $\tau^{\mathrm{order}}\left(A_{j}^{i,r}\right)$ by regressing the outcome characterized by equation (1) on attributes of interest with implementing simple OLS fixing the intercept at 0. Since we convert all the background characteristics to binary dummy variables for analysis, our estimates are marginal means (Hainmueller et al., 2014; Leeper et al., 2020).

As described in 2.4.1, one out of the three hypothetical recipients is always a Japanese citizen. This design is used to identify which attributes lead Japanese respondents to prioritize a foreign recipient over a Japanese recipient. Thus, another outcome of interest is $Y_{i,j,r}^{\mathrm{FoverJ}}$, which takes a value of 1 if and only if respondent *i* prioritizes a foreign recipient with attributes $A_{F,j}^{i,r}$ over a Japanese recipient with attributes $A_{J,j^{\prime }}^{i,r}$ in round *r*, where attributes other than the citizenship of $A_{F,j}^{i,r}$ and $A_{J,j^{\prime }}^{i,r}$ are allowed to be randomly assigned to be either equivalent or different, such that

(3) $$Y_{i,j,r}^{\mathrm{FoverJ}}\left(A_{F,j}^{i,r},A_{J,j^{\prime }}^{i,r}\right)=\left\{\begin{array}{cc} 1 & \text{if}\;A_{F,j}^{i,r}{≻}_{i}A_{J,j^{\prime }}^{i,r},\\ 0 & \text{if}\;A_{F,j}^{i,r}{≺}_{i}A_{J,j^{\prime }}^{i,r}. \end{array}\right)$$

We evaluate the respondents’ prioritization of foreign recipients over Japanese recipients by estimating the average marginal mean, with Japanese recipients as the reference point, such that

(4) $$\tau^{\mathrm{FoverJ}}\left(A_{F,j}^{i,r},A_{J,j^{\prime }}^{i,r}\right)=E\left[Y_{j}^{\mathrm{FoverJ}}\left(A_{F,j}^{i,r},A_{J,j^{\prime }}^{i,r}\right)\right].$$

We estimate $\tau^{\mathrm{FoverJ}}\left(A_{F,j}^{i,r},A_{J,j^{\prime }}^{i,r}\right)$ by regressing the outcome characterized by equation (3) on attributes of interest with implementing simple OLS regression fixing the intercept at 0. As described above, since all the analyzed background characteristics are converted to dummy variables, our estimates are marginal means (Hainmueller et al., 2014; Leeper et al., 2020).

Suppose that *a*_*j*,*l*_ is the *l*th attribute of hypothetical recipient ***A***_*j*_. Then, since *a*_*j*,*l*_ is randomly drawn, *a*_*j*,*l*_ satisfies the unconfounded assumption,

$$a_{j,l}⫫Y\left(A_{j}\right).$$

Thus, we identify $\tau^{\mathrm{order}}\left(A_{j}^{i,r}\right)$ characterized by equation (2) and $\tau^{\mathrm{FoverJ}}\left(A_{F,j}^{i,r},A_{J,j^{\prime }}^{i,r}\right)$ by equation (4) as causal effects of $A_{j}^{i,r}$ and the sets $A_{F,j}^{i,r}$ and $A_{J,j^{\prime }}^{i,r}$, respectively.

In our estimation, we focus on the marginal value of the outcome given a level of attribute *l* as follows:

(5) $$\sum_{A_{-lj},A_{j^{'}},A_{j^{''}}}E\left[Y_{i}\left(A_{j},A_{-lj},A_{j^{'}},A_{j^{''}}\right)\right]\times f\left(A_{j},A_{-lj},A_{j^{'}},A_{j^{''}}\right),$$

and

(6) $$\sum_{A_{F,-lj},A_{J,j^{'}}}E\left[Y_{i}\left(A_{F,j},A_{F,-lj},A_{J,j^{'}}\right)\right]\times f\left(A_{F,j},A_{F,-lj},A_{J,j^{'}}\right),$$

where ${Α}_{-\text{l}j}$ denotes the vector created by removing element *l* from ${Α}_{j}$, and *f* denotes the joint density function.

### 3.2 Billed at cost or subsidized

Next, let $D_{i}^{r}\in \left\{1,2\right\}$ be the scenario shown to respondent *i* in round *r*, as described in section 2.4.2 above such that $D_{i}^{r}=1$ denotes “billed at cost” and $D_{i}^{r}=2$ denotes “government subsidized.” We are interested in whether the responsibility for payment affects the respondents’ prioritization of foreign recipients over Japanese recipients. Because the observed $A_{F,j}^{i,r}$ and $D_{i}^{r}$ are randomized, the average potential marginal outcome is identified as follows:

(7) $$E\left[Y_{i}^{\mathrm{FoverJ}}\left(A_{F,j}^{i,r},A_{J,j^{\prime }}^{i,r},d\right)\right]=E\left[Y_{i}^{\mathrm{FoverJ}}|A_{F,j}^{i,r},A_{J,j^{\prime }}^{i,r},D_{i}^{r}=d\right].$$

Since $D_{i}^{r}$ is randomly drawn, $D_{i}^{r}$ satisfies the unconfounded assumption

$$D_{i}^{r}⫫Y_{i}^{\mathrm{FoverJ}}\left(A_{F,j}^{i,r},A_{J,j^{'}}^{i,r}D_{i}^{r}=d\right),$$

so we can identify

(8) $$E\left[Y_{i}^{\mathrm{FoverJ}}\left(A_{F,j}^{i,r},A_{J,j^{'}}^{i,r},D_{i}=2\right)-Y_{i}^{\mathrm{FoverJ}}\left(A_{F,j}^{i,r},A_{J,j^{'}}^{i,r},D_{i}=1\right)\right]$$

as the causal effect of subsidizing vaccination by the government (*D*_*i*_ = 2) relative to billing at cost (*D*_*i*_ = 1).

## 4 Results

### 4.1 Effects of treatment 1: Average marginal expected means

**Fig. 1:**
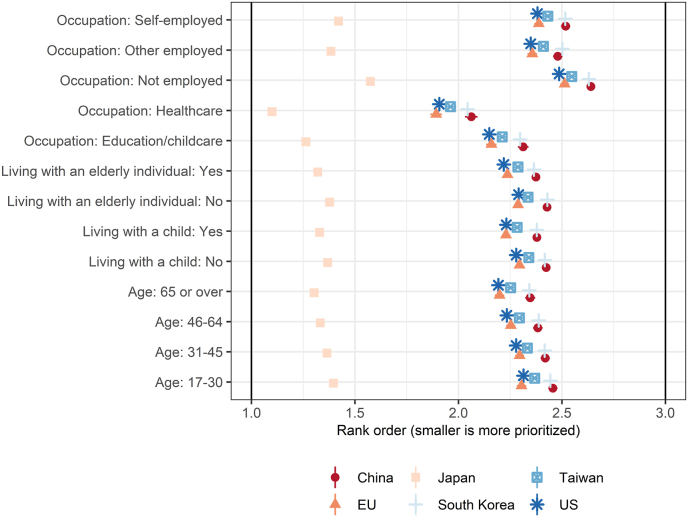
Foreigner penalty in COVID-19 vaccine deployment. *Notes*: The 95% clustering robust confidence interval without multiple testing adjustment is shown. Since confidence intervals are short, they are shaded by shapes. Point estimates and confidence intervals are reported in Table A1 in the Appendix.

Fig. 1 presents the order of prioritization as characterized by equation (2) as $\tau^{\mathrm{order}}\left(A_{j}^{i,r}\right)$ over all other attribute combinations than citizenship. Since one member out of each group of three hypothetical recipients is always a Japanese citizen, the results ultimately capture the penalty on foreigners. The horizontal axis denotes the order of prioritization in our conjoint design such that 3 indicates the lowest prioritization as described by equation (1). Therefore, a higher estimate implies lower priority. On average, respondents prioritized Japanese citizens over foreigners to a substantial degree across visa types, citizenship status, occupation, demographics, and family risk characteristics, such as living with an elderly individual or a child. The prioritization of fellow citizens is deeply rooted, which is consistent with the results of Knotz et al. (2021b). Since we set up scenarios in which there was a shortage of vaccines, we interpret this citizenship prioritization result as due to concerns about the allocation of scarce resources, as in the ICU triage case discussed in Knotz et al. (2021b).

Among foreigners, American, European, and Taiwanese citizens received higher priority over Chinese and South Korean citizens. We interpret this result as indicating the effect of prepandemic geopolitical concerns with China and South Korea.

Fig. 1 presents the average responses of Japanese respondents. We cannot identify the effects of individual attributes on responses, which might not be negligible, with average marginal expected means shown in Fig. 1.

**Fig. 2:**
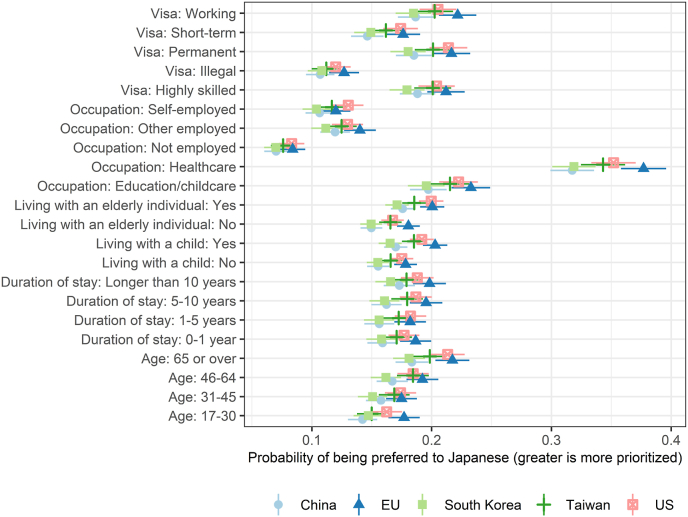
Probability of foreigners being prioritized for COVID-19 vaccine deployment. *Notes*: The 95% clustering robust confidence interval without multiple testing adjustment is shown. Point estimates and confidence intervals are reported in Table A2 in the Appendix.

Therefore, Fig. 2 depicts the probability that foreigners were prioritized over Japanese citizens according to their background characteristics related to occupation, visa type, family composition, duration of stay in Japan, and age, as characterized by equation (4), $\tau^{\mathrm{FoverJ}}\left(A_{F,j}^{i,r},A_{J,j^{\prime }}^{i,r}\right)$.

The horizontal axis denotes the probability that the foreign candidate recipients were prioritized over Japanese candidate recipients on average. The results show that if the foreigners were healthcare workers, they were prioritized over Japanese citizens with a probability over 30% on average. If the foreigners were education or childcare workers and American, European, or Taiwanese citizens, they were prioritized over Japanese citizens with a probability higher than 20%. We interpret these results as showing that healthcare workers and teachers were prioritized because the positive externalities of their occupation amplified the positive externalities associated with vaccination. Foreigners with working, permanent, or highly skilled visas or those aged 65 or over were also prioritized over Japanese citizens with a probability of approximately 20%. Thus foreigners exposed to greater risk were considered more deserving of vaccination. If the positive externality of herd immunity was considered, foreigners with a longer duration of stay would be considered more deserving of vaccination. The probability increases linearly with the duration of stay. Therefore, the result is not inconsistent with our expectation of positive externality.

Note that Fig. 2 presents marginal means across all attributes and does not show marginal means when specific attributes are fixed. For instance, Fig. 2 does not show marginal means when occupations are fixed. Thus, Fig. 2 does not tell whether the probability of foreigners being prioritized over Japanese citizens is still higher if both foreigners and Japanese nationals are healthcare workers or education/childcare workers.

**Fig. 3:**
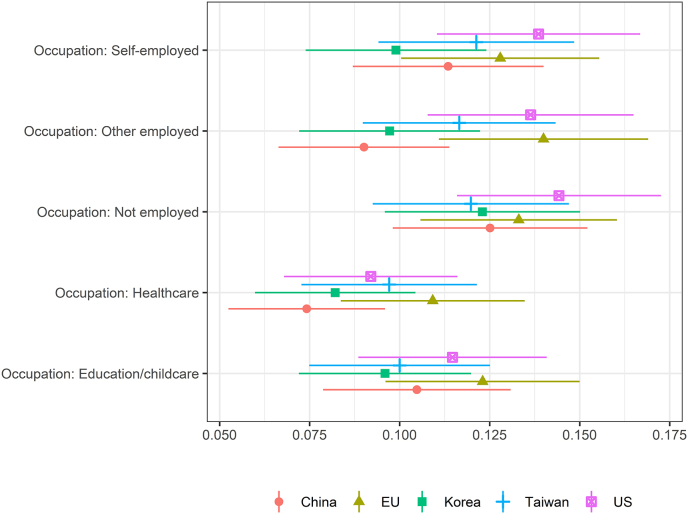
Probability of foreigners being prioritized for COVID-19 vaccine deployment when the occupations of foreign and Japanese candidates are the same. *Notes*: The 95% clustering robust confidence interval without multiple testing adjustment is shown. Point estimates and confidence intervals are reported in Table A3 in the Appendix.

The answer is No. Fig. 3 presents $\tau^{\mathrm{FoverJ}}\left(A_{F,j}^{i,r},A_{J,j^{\prime }}^{i,r}\right)$ by equation (4) when the occupations of hypothetical foreign and Japanese recipients were the same. When both foreign and Japanese candidates were healthcare workers and when both foreign and Japanese candidates were educational/childcare workers, the probability of foreigners being prioritized over Japanese citizens was lower than otherwise. In summary, respondents prioritized healthcare, educational, and childcare workers in general, and when both foreigners and Japanese were healthcare workers or educational/childcare workers, Japanese candidates were prioritized. Occupational positive externality raised the probability of foreigners being prioritized over Japanese but did not weaken citizen priority itself.

**Fig. 4:**
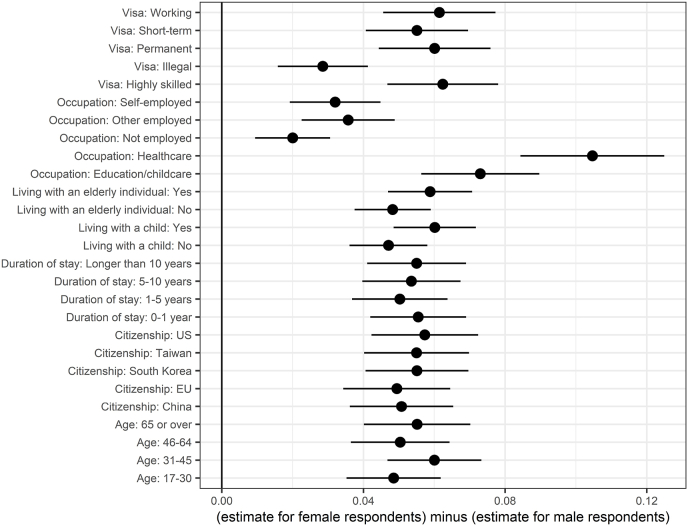
Gender differences in the probability of prioritizing foreign rather than Japanese recipients. *Notes*: The 95% clustering robust confidence interval without multiple testing adjustment is shown. Point estimates and confidence intervals are reported in Table A4 in the Appendix.

Additionally, although the effects were qualitatively similar across respondents with various background characteristics in Fig. 2, they could be heterogeneous in terms of extent. Let us identify possible differences by gender as a typical case of heterogeneity. Fig. 4 presents the difference in the probability of prioritizing foreign recipients over Japanese recipients by respondents’ gender $\underset{i_{f},i_{m}}{\Delta }\tau^{\mathrm{FoverJ}}\left(A_{F,j}^{i,r},A_{J,j^{\prime }}^{i,r}\right)$ such that

(9) $$\underset{i_{f},i_{m}}{\Delta }\tau^{\mathrm{FoverJ}}\left(A_{F,j}^{i,r},A_{J,j^{\prime }}^{i,r}\right)\equiv \tau^{\mathrm{FoverJ}}\left(A_{F,j}^{i_{f},r},A_{J,j^{\prime }}^{i_{f},r}\right)-\tau^{\mathrm{FoverJ}}\left(A_{F,j}^{i_{m},r},A_{J,j^{\prime }}^{i_{m},r}\right),$$

where *i*_*f*_ and *i*_*m*_ denote female and male respondents, respectively, and $\tau^{\mathrm{FoverJ}}\left(A_{F,j}^{i_{f},r},A_{J,j^{\prime }}^{i_{f},r}\right)$ and $\tau^{\mathrm{FoverJ}}\left(A_{F,j}^{i_{m},r},A_{J,j^{\prime }}^{i_{m},r}\right)$ denote the probability that female respondents and male respondents, respectively, prioritize foreign recipients over Japanese recipients as characterized by equation (4).

Thus, $\underset{i_{f},i_{m}}{\Delta }\tau^{\mathrm{FoverJ}}\left(A_{F,j}^{i,r},A_{J,j^{\prime }}^{i,r}\right)$ measures the extent to which female respondents are more likely to prioritize foreign recipients over Japanese recipients than male respondents were. A positive estimate means that female respondents were more likely to prioritize foreign recipients over Japanese recipients than male respondents. Across recipient attributes, female respondents were more likely to prioritize foreign recipients than male respondents. Of the attributes, occupation elicited the most significant gender differences. The degree to which healthcare and educational workers were prioritized was substantially higher among female respondents than among male respondents. In summary, female respondents’ foreigner penalty was smaller than male respondents’ across recipient attributes, and female respondents valued the positive occupational externalities of doctors, nurses, and teachers more highly.

### 4.2 Effects of treatment 2: billed at cost or government-subsidized

**Fig. 5:**
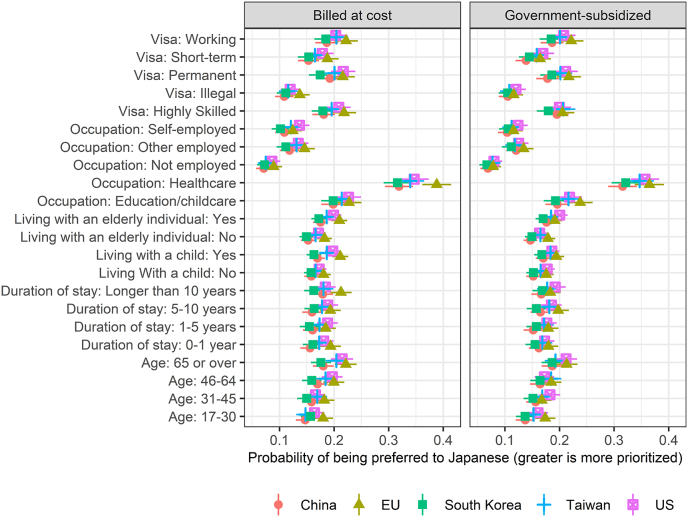
Two scenarios: Billed at cost or government subsidized. *Notes*: The 95% clustering robust confidence interval without multiple testing adjustment is shown. Point estimates and confidence intervals are shown in Table A5 of the Appendix.

We did not find a significant difference between whether vaccination was billed at cost or government subsidized, characterized by equation (8). Fig. 5 presents the predicted probability of prioritizing foreign over Japanese recipients $\tau^{\mathrm{FoverJ}}\left(A_{F,j}^{i,r},A_{J,j^{\prime }}^{i,r}\right)$, characterized by equation (4) in treatment 2 in section 2.4.2. That is, $\tau^{\mathrm{FoverJ}}\left(A_{F,j}^{i,r},A_{J,j^{\prime }}^{i,r}\right)$ is presented for cases in which vaccination is billed at cost and for cases in which it is government subsidized. Being billed at cost or government subsidized did not substantially affect the probability that respondents prioritized a foreign recipient over a Japanese recipient on average, relative to the differences shown in Fig. 2.

Therefore, the source of funding for vaccination was statistically irrelevant to the penalty on foreigners and the prioritization of healthcare and educational workers, including foreigners, described in 4.1. Neither exclusion-oriented attitudes toward foreigners nor inclusion-oriented attitudes toward workers with large positive occupational externalities were statistically associated with the funding source. Note that the question shown to respondents assumed scarcity of COVID-19 vaccines. If concerns about scarcity were large enough, we could not have identified any concerns about state subsidies to vaccinate foreigners. In other words, our result does not imply that Japanese citizens are generous about funding vaccination for foreigners in general.

## 5 Conclusions

Our results show that the Japanese prioritize Japanese candidates for vaccine receipt over foreign candidate recipients. The finding that fellow citizens are prioritized in the allocation of scarce resources is consistent with the findings of previous works such as Reeskens et al. (2021), Vinay et al. (2021), Knotz et al. (2021b), and Larsen and Schaeffer (2021). Additionally, our results indicate that geopolitical concerns impose a penalty on foreigners. Such exclusionist attitudes are irrelevant to whether vaccination is billed at cost or subsidized by the government.

However, our results also show that positive occupational externalities, such as those associated with healthcare and education, mitigate the penalty on foreigners. A substantial portion of Japanese respondents indicated that such foreigners should be prioritized over Japanese citizens on average. The effects were qualitatively similar across respondent background characteristics, but the magnitudes of the effects were heterogeneous. As a typical example, we found that between female and male respondents, female respondents imposed substantially smaller foreign penalties such that the probability that female respondents prioritized foreign over Japanese recipients was substantially higher than that of male respondents across recipient attributes. Furthermore, female respondents exhibited a substantially higher probability of prioritizing foreign over Japanese recipients if the recipients’ occupation was healthcare or education. Women are more inclusive on average and value positive occupational externalities more than men.

Previous works on vaccine hesitancy, such as Kreps et al. (2020); Motta (2021); Niño et al. (2021); Schwarzinger et al. (2021); Kawata and Nakabayashi (2021); Hara et al. (2021); Baccolini et al. (2021); Latkin et al. (2021); Niu et al. (2022), Stöckli et al. (2022), Falcone et al. (2022), among others, have addressed the interference of vaccine hesitancy in the positive externalities associated with vaccination, implicitly assuming that vaccines are adequately supplied. In the sense that we focus on the positive externalities of vaccination, we share an interest with these works.

However, we also share a research interest in the fair allocation of scarce medical resources with Reeskens et al. (2021), O’Dell et al. (2019), Wall et al. (2020), Reeskens et al. (2021), Vinay et al. (2021), and Knotz et al. (2021b), among others. Our unique contribution is our focus on the possible trade-off between citizens’ priorities and positive medical externalities to identify whether positive medical externalities, amplified by the positive occupational externalities of recipients, can mitigate the exclusionist attitudes of citizens. Our results show that they can.

Despite having exclusionist attitudes toward foreigners regarding the allocation of scarce resources, a nonnegligible portion of Japanese citizens understand that foreigners in occupations with large positive occupational externalities should be prioritized over Japanese citizens on average. Our results have a straightforward policy implication. Currently, the Japanese government operates two channels for the provision of vaccination against COVID-19: through municipal governments and at workplaces. Since our results indicate that Japanese citizens have the most inclusionist attitudes regarding vaccination toward those in healthcare and education, the government is advised to explicitly prioritize healthcare and education when allocating vaccines through workplace vaccination channels if vaccine shortages are severe because such actions would be supported by a substantial portion of Japanese citizens.

While we consider our results indicate respondents’ appreciation of the positive externality of healthcare and educational workers, we admit that other interpretations are possible. Humans are inclined to heuristically judge deservingness by simplified signals of reciprocity to mutually help by a lighter cognitive load, which is referred to as the “deservingness heuristic” (Gandenberger et al., 2022; Petersen, 2012; van Oorschot, 2000). Healthcare, educational, and childcare workers’ jobs are supportive of others. This impression of their occupations might be valued from the viewpoint of reciprocity. Although the positive externality would encourage reciprocal behaviors, reciprocal behaviors do not necessarily accompany the positive externality. The recognition of positive externality and reciprocity scenarios are not mutually exclusive, but the latter does not imply the former because people could behave reciprocally in return even if the interactive behaviors do not have positive externalities. Therefore, our interpretation of the positive externality is based on a stronger assumption than reciprocity is. Furthermore, we assume that respondents took on a greater cognitive load than required to identify the deservingness heuristic. While we admit that the deservingness heuristic is a plausible candidate to interpret our results, we leave it for future research to identify which is more plausible.

Also, the higher perceived deservingness of older foreigners in Fig. 2 cannot be explained only by consideration of the positive externality. A more straightforward interpretation would be altruism. While altruism is a driving force of protective behaviors (Cato et al., 2020), a challenge is that the pandemic has tended to direct altruistic attitudes toward local communities (Grimalda et al., 2021). Our results show that altruistic attitudes could still accommodate foreigners. This indicates the possibility that altruism may not be limited to homogeneous local communities.

Additionally, our finding that exclusionist attitudes toward foreigners are mitigated by positive occupational externalities reminds us of crossed categorization, which refers to situations in which interactions between different categories such as citizenship, ethnicity, occupation, race, or religion might mitigate exclusionist attitudes toward outsiders, as defined in terms of one of the categories of interest (Crisp & Hewstone, 2007; Deschamps & Doise, 1978; Grigoryan, 2020; Prati et al., 2021). Since the design of our background characteristics survey does not allow us to evaluate cases in which Japanese respondents’ own attributes other than citizenship, such as occupation or living with an elderly individual, are the same as or different from those of foreigners, we cannot directly compare our results with results based on the crossed-categorization hypothesis. However, another conjoint design to evaluate the crossed-categorization hypothesis would be surely an attractive project for future research.

## Conflicts of interest, ethical review, and preregistration

● The authors declare that they have no relevant conflicts of interest that relate to the research described in this paper.
● The Ethical Review Board of the Institute of Social Science, The University of Tokyo approved this study (Approval Number: 73).
● The design and projected outcomes were preregistered with the AEA RCT Registry (RCT ID: AEARCTR-0008105, Iida et al. (2022)).

## Figures and Tables

**Table A1:** Estimated ranking of hypothetical vaccine recipients (Fig. 1).

Confidence low	Confidence high	Estimate	Attributes	Citizenship status
1.09	1.11	1.10	Occupation: Healthcare	Japan
1.25	1.28	1.26	Occupation: Education	Japan
1.37	1.40	1.38	Occupation: Other employed	Japan
1.40	1.44	1.42	Occupation: Self-employed	Japan
1.55	1.60	1.57	Occupation: Not employed	Japan
1.38	1.41	1.40	Age: 17–30	Japan
1.35	1.38	1.37	Age: 31–45	Japan
1.32	1.35	1.33	Age: 46–64	Japan
1.29	1.32	1.30	Age: 65 or over	Japan
1.32	1.34	1.33	Living with a child: Yes	Japan
1.36	1.38	1.37	Living with a child: No	Japan
1.31	1.33	1.32	Living with an elderly individual: Yes	Japan
1.36	1.39	1.38	Living with an elderly individual: No	Japan
1.88	1.93	1.91	Occupation: Healthcare	US
2.12	2.17	2.15	Occupation: Education	US
2.33	2.37	2.35	Occupation: Other employed	US
2.36	2.40	2.38	Occupation: Self-employed	US
2.46	2.51	2.49	Occupation: Not employed	US
2.29	2.34	2.31	Age: 17–30	US
2.26	2.30	2.28	Age: 31–45	US
2.21	2.25	2.23	Age: 46–64	US
2.17	2.22	2.19	Age: 65 or over	US
2.22	2.25	2.23	Living with a child: Yes	US
2.26	2.29	2.28	Living with a child: No	US
2.20	2.23	2.22	Living with an elderly individual: Yes	US
2.28	2.31	2.29	Living with an elderly individual: No	US
2.03	2.09	2.06	Occupation: Healthcare	China
2.29	2.34	2.31	Occupation: Education	China
2.46	2.50	2.48	Occupation: Other employed	China
2.50	2.54	2.52	Occupation: Self-employed	China
2.62	2.66	2.64	Occupation: Not employed	China
2.43	2.48	2.46	Age: 17–30	China
2.40	2.44	2.42	Age: 31–45	China
2.36	2.41	2.38	Age: 46–64	China
2.32	2.37	2.35	Age: 65 or over	China
2.36	2.40	2.38	Living with a child: Yes	China
2.41	2.44	2.42	Living with a child: No	China
2.36	2.39	2.37	Living with an elderly individual: Yes	China
2.41	2.44	2.43	Living with an elderly individual: No	China
1.93	1.99	1.96	Occupation: Healthcare	Taiwan
2.19	2.24	2.21	Occupation: Education	Taiwan
2.39	2.43	2.41	Occupation: Other employed	Taiwan
2.41	2.45	2.43	Occupation: Self-employed	Taiwan
2.53	2.57	2.55	Occupation: Not employed	Taiwan
2.35	2.39	2.37	Age: 17–30	Taiwan
2.31	2.35	2.33	Age: 31–45	Taiwan
2.27	2.32	2.29	Age: 46–64	Taiwan
2.23	2.28	2.25	Age: 65 or over	Taiwan
2.27	2.30	2.28	Living with a child: Yes	Taiwan
2.32	2.36	2.34	Living with a child: No	Taiwan
2.27	2.30	2.29	Living with an elderly individual: Yes	Taiwan
2.32	2.35	2.34	Living with an elderly individual: No	Taiwan
2.01	2.07	2.04	Occupation: Healthcare	South Korea
2.27	2.32	2.30	Occupation: Education	South Korea
2.48	2.52	2.50	Occupation: Other employed	South Korea
2.49	2.54	2.52	Occupation: Self-employed	South Korea
2.61	2.65	2.63	Occupation: Not employed	South Korea
2.42	2.47	2.44	Age: 17–30	South Korea
2.39	2.44	2.42	Age: 31–45	South Korea
2.37	2.41	2.39	Age: 46–64	South Korea
2.32	2.37	2.34	Age: 65 or over	South Korea
2.36	2.39	2.38	Living with a child: Yes	South Korea
2.40	2.43	2.42	Living with a child: No	South Korea
2.35	2.38	2.36	Living with an elderly individual: Yes	South Korea
2.41	2.45	2.43	Living with an elderly individual: No	South Korea
1.86	1.92	1.89	Occupation: Healthcare	EU
2.13	2.18	2.16	Occupation: Education	EU
2.33	2.38	2.36	Occupation: Other employed	EU
2.37	2.41	2.39	Occupation: Self-employed	EU
2.49	2.53	2.51	Occupation: Not employed	EU
2.28	2.33	2.30	Age: 17–30	EU
2.27	2.32	2.30	Age: 31–45	EU
2.23	2.27	2.25	Age: 46–64	EU
2.18	2.22	2.20	Age: 65 or over	EU
2.21	2.25	2.23	Living with a child: Yes	EU
2.28	2.31	2.30	Living with a child: No	EU
2.22	2.25	2.24	Living with an elderly individual: Yes	EU
2.27	2.30	2.29	Living with an elderly individual: No	EU

**Table A2:** Estimated probability of being prioritized over Japanese citizens (Fig. 2).

Confidence low	Confidence high	Estimate	Attributes	Citizenship status
0.33	0.37	0.35	Occupation: Healthcare	US
0.21	0.24	0.22	Occupation: Education	US
0.12	0.14	0.13	Occupation: Other employed	US
0.12	0.14	0.13	Occupation: Self-employed	US
0.07	0.09	0.08	Occupation: Not employed	US
0.15	0.18	0.16	Age: 17–30	US
0.16	0.19	0.17	Age: 31–45	US
0.17	0.20	0.18	Age: 46–64	US
0.20	0.23	0.21	Age: 65 or over	US
0.18	0.20	0.19	Living with a child: Yes	US
0.17	0.18	0.18	Living with a child: No	US
0.19	0.21	0.20	Living with an elderly individual: Yes	US
0.16	0.18	0.17	Living with an elderly individual: No	US
0.16	0.19	0.18	Duration of stay: 0–1 year	US
0.17	0.20	0.18	Duration of stay: 1–5 years	US
0.17	0.20	0.19	Duration of stay: 5–10 years	US
0.17	0.20	0.19	Duration of stay: 10 years or longer	US
0.16	0.19	0.17	Visa: Short-term	US
0.19	0.22	0.20	Visa: Highly skilled	US
0.19	0.22	0.21	Visa: Working	US
0.20	0.23	0.21	Visa: Permanent	US
0.11	0.13	0.12	Visa: Illegal	US
0.30	0.34	0.32	Occupation: Healthcare	China
0.18	0.21	0.20	Occupation: Education	China
0.11	0.13	0.12	Occupation: Other employed	China
0.09	0.12	0.11	Occupation: Self-employed	China
0.06	0.08	0.07	Occupation: Not employed	China
0.13	0.15	0.14	Age: 17–30	China
0.15	0.17	0.16	Age: 31–45	China
0.15	0.18	0.17	Age: 46–64	China
0.17	0.20	0.18	Age: 65 or over	China
0.16	0.18	0.17	Living with a child: Yes	China
0.15	0.16	0.16	Living with a child: No	China
0.17	0.19	0.18	Living with an elderly individual: Yes	China
0.14	0.16	0.15	Living with an elderly individual: No	China
0.15	0.17	0.16	Duration of stay: 0–1 year	China
0.14	0.17	0.16	Duration of stay: 1–5 years	China
0.15	0.18	0.16	Duration of stay: 5–10 years	China
0.16	0.19	0.17	Duration of stay: 10 years or longer	China
0.13	0.16	0.15	Visa: Short-term	China
0.17	0.20	0.19	Visa: Highly skilled	China
0.17	0.20	0.19	Visa: Working	China
0.17	0.20	0.19	Visa: Permanent	China
0.10	0.12	0.11	Visa: Illegal	China
0.32	0.36	0.34	Occupation: Healthcare	Taiwan
0.20	0.23	0.22	Occupation: Education	Taiwan
0.11	0.14	0.12	Occupation: Other employed	Taiwan
0.10	0.13	0.12	Occupation: Self-employed	Taiwan
0.07	0.09	0.08	Occupation: Not employed	Taiwan
0.14	0.16	0.15	Age: 17–30	Taiwan
0.16	0.18	0.17	Age: 31–45	Taiwan
0.17	0.20	0.18	Age: 46–64	Taiwan
0.18	0.21	0.20	Age: 65 or over	Taiwan
0.18	0.20	0.19	Living with a child: Yes	Taiwan
0.16	0.18	0.17	Living with a child: No	Taiwan
0.18	0.20	0.19	Living with an elderly individual: Yes	Taiwan
0.16	0.17	0.17	Living with an elderly individual: No	Taiwan
0.16	0.18	0.17	Duration of stay: 0–1 year	Taiwan
0.16	0.19	0.17	Duration of stay: 1–5 years	Taiwan
0.17	0.19	0.18	Duration of stay: 5–10 years	Taiwan
0.17	0.19	0.18	Duration of stay: 10 years or longer	Taiwan
0.15	0.18	0.16	Visa: Short-term	Taiwan
0.19	0.22	0.20	Visa: Highly skilled	Taiwan
0.19	0.22	0.20	Visa: Working	Taiwan
0.19	0.22	0.20	Visa: Permanent	Taiwan
0.10	0.12	0.11	Visa: Illegal	Taiwan
0.30	0.34	0.32	Occupation: Healthcare	South Korea
0.18	0.21	0.20	Occupation: Education	South Korea
0.10	0.12	0.11	Occupation: Other employed	South Korea
0.09	0.12	0.10	Occupation: Self-employed	South Korea
0.06	0.08	0.07	Occupation: Not employed	South Korea
0.13	0.16	0.15	Age: 17–30	South Korea
0.14	0.16	0.15	Age: 31–45	South Korea
0.15	0.17	0.16	Age: 46–64	South Korea
0.17	0.19	0.18	Age: 65 or over	South Korea
0.16	0.17	0.17	Living with a child: Yes	South Korea
0.15	0.16	0.16	Living with a child: No	South Korea
0.16	0.18	0.17	Living with an elderly individual: Yes	South Korea
0.14	0.16	0.15	Living with an elderly individual: No	South Korea
0.15	0.17	0.16	Duration of stay: 0–1 year	South Korea
0.14	0.17	0.16	Duration of stay: 1–5 years	South Korea
0.15	0.17	0.16	Duration of stay: 5–10 years	South Korea
0.15	0.18	0.17	Duration of stay: 10 years or longer	South Korea
0.14	0.16	0.15	Visa: Short-term	South Korea
0.16	0.19	0.18	Visa: Highly skilled	South Korea
0.17	0.20	0.18	Visa: Working	South Korea
0.17	0.20	0.18	Visa: Permanent	South Korea
0.10	0.12	0.11	Visa: Illegal	South Korea
0.36	0.40	0.38	Occupation: Healthcare	EU
0.22	0.25	0.23	Occupation: Education	EU
0.13	0.15	0.14	Occupation: Other employed	EU
0.11	0.13	0.12	Occupation: Self-employed	EU
0.07	0.09	0.08	Occupation: Not employed	EU
0.16	0.19	0.18	Age: 17–30	EU
0.16	0.19	0.17	Age: 31–45	EU
0.18	0.21	0.19	Age: 46–64	EU
0.20	0.23	0.22	Age: 65 or over	EU
0.19	0.21	0.20	Living with a child: Yes	EU
0.17	0.19	0.18	Living with a child: No	EU
0.19	0.21	0.20	Living with an elderly individual: Yes	EU
0.17	0.19	0.18	Living with an elderly individual: No	EU
0.17	0.20	0.19	Duration of stay: 0–1 year	EU
0.17	0.20	0.18	Duration of stay: 1–5 years	EU
0.18	0.21	0.20	Duration of stay: 5–10 years	EU
0.18	0.21	0.20	Duration of stay: 10 years or longer	EU
0.16	0.19	0.18	Visa: Short-term	EU
0.20	0.23	0.21	Visa: Highly skilled	EU
0.21	0.24	0.22	Visa: Working	EU
0.20	0.23	0.22	Visa: Permanent	EU
0.11	0.14	0.13	Visa: Illegal	EU

**Table A3:** Estimated probability of being prioritized over Japanese citizens when foreign and Japanese candidates’ occupation is the same (Fig. 3).

Confidence low	Confidence high	Estimate	Citizenship	Occupation
0.09	0.07	0.12	US	Occupation: Healthcare
0.11	0.09	0.14	US	Occupation: Education/childcare
0.14	0.11	0.16	US	Occupation: Other employed
0.14	0.11	0.17	US	Occupation: Self-employed
0.14	0.12	0.17	US	Occupation: Not employed
0.07	0.05	0.10	China	Occupation: Healthcare
0.10	0.08	0.13	China	Occupation: Education/childcare
0.09	0.07	0.11	China	Occupation: Other employed
0.11	0.09	0.14	China	Occupation: Self-employed
0.13	0.10	0.15	China	Occupation: Not employed
0.10	0.07	0.12	Taiwan	Occupation: Healthcare
0.10	0.07	0.13	Taiwan	Occupation: Education/childcare
0.12	0.09	0.14	Taiwan	Occupation: Other employed
0.12	0.09	0.15	Taiwan	Occupation: Self-employed
0.12	0.09	0.15	Taiwan	Occupation: Not employed
0.08	0.06	0.10	Korea	Occupation: Healthcare
0.10	0.07	0.12	Korea	Occupation: Education/childcare
0.10	0.07	0.12	Korea	Occupation: Other employed
0.10	0.07	0.12	Korea	Occupation: Self-employed
0.12	0.10	0.15	Korea	Occupation: Not employed
0.11	0.08	0.13	EU	Occupation: Healthcare
0.12	0.10	0.15	EU	Occupation: Education/childcare
0.14	0.11	0.17	EU	Occupation: Other employed
0.13	0.10	0.16	EU	Occupation: Self-employed
0.13	0.11	0.16	EU	Occupation: Not employed
				

**Table A4:** Differences in the probability of prioritizing foreign recipients over Japanese recipients by respondent gender (Fig. 4)

Confidence low	Confidence high	Estimate	Attributes
0.08	0.12	0.10	Occupation: Healthcare
0.06	0.09	0.07	Occupation: Education
0.02	0.05	0.04	Occupation: Other employed
0.02	0.04	0.03	Occupation: Self-employed
0.01	0.03	0.02	Occupation: Not employed
0.04	0.07	0.06	Citizenship: US
0.04	0.07	0.05	Citizenship: China
0.04	0.07	0.06	Citizenship: Taiwan
0.04	0.07	0.06	Citizenship: South Korea
0.03	0.06	0.05	Citizenship: EU
0.04	0.06	0.05	Age: 17–30
0.05	0.07	0.06	Age: 31–45
0.04	0.06	0.05	Age: 46–64
0.04	0.07	0.06	Age: 65 or over
0.05	0.07	0.06	Living with a child: Yes
0.04	0.06	0.05	Living with a child: No
0.05	0.07	0.06	Living with an elderly individual: Yes
0.04	0.06	0.05	Living with an elderly individual: No
0.04	0.07	0.06	Duration of stay: 0–1 year
0.04	0.06	0.05	Duration of stay: 1–5 years
0.04	0.07	0.05	Duration of stay: 5–10 years
0.04	0.07	0.06	Duration of stay: 10 years or over
0.04	0.07	0.06	Visa: Short-term
0.05	0.08	0.06	Visa: Highly skilled
0.05	0.08	0.06	Visa: Working
0.04	0.08	0.06	Visa: Permanent
0.02	0.04	0.03	Visa: Illegal
			

**Table A5:** Estimated probability of foreigners being prioritized over Japanese recipients on average under two payment scenarios: Billed at cost or government subsidized (Fig. 5).

Confidence low	confidence high	Estimate	Attributes	Citizenship status	Scenario
0.32	0.37	0.35	Occupation: Healthcare	US	Billed at cost
0.20	0.25	0.23	Occupation: Education/childcare	US	Billed at cost
0.12	0.15	0.13	Occupation: Other employed	US	Billed at cost
0.12	0.15	0.14	Occupation: Self-employed	US	Billed at cost
0.07	0.10	0.09	Occupation: Not employed	US	Billed at cost
0.15	0.18	0.16	Age: 17-30	US	Billed at cost
0.15	0.18	0.17	Age: 31-45	US	Billed at cost
0.18	0.22	0.20	Age: 46-64	US	Billed at cost
0.19	0.23	0.21	Age: 65 or over	US	Billed at cost
0.18	0.21	0.20	Living with a child: Yes	US	Billed at cost
0.16	0.19	0.17	Living With a child: No	US	Billed at cost
0.18	0.21	0.20	Living with an elderly individual: Yes	US	Billed at cost
0.16	0.18	0.17	Living with an elderly individual: No	US	Billed at cost
0.16	0.20	0.18	Duration of stay: 0–1 year	US	Billed at cost
0.17	0.21	0.19	Duration of stay: 1–5 years	US	Billed at cost
0.17	0.21	0.19	Duration of stay: 5–10 years	US	Billed at cost
0.16	0.20	0.18	Duration of stay: Longer than 10 years	US	Billed at cost
0.16	0.20	0.18	Visa: Short-term	US	Billed at cost
0.19	0.23	0.21	Visa: Highly skilled	US	Billed at cost
0.18	0.22	0.20	Visa: Working	US	Billed at cost
0.19	0.24	0.22	Visa: Permanent	US	Billed at cost
0.10	0.14	0.12	Visa: Illegal	US	Billed at cost
0.29	0.34	0.32	Occupation: Healthcare	China	Billed at cost
0.18	0.22	0.20	Occupation: Education/childcare	China	Billed at cost
0.10	0.14	0.12	Occupation: Other employed	China	Billed at cost
0.09	0.13	0.11	Occupation: Self-employed	China	Billed at cost
0.06	0.09	0.07	Occupation: Not employed	China	Billed at cost
0.13	0.16	0.15	Age: 17-30	China	Billed at cost
0.14	0.18	0.16	Age: 31-45	China	Billed at cost
0.15	0.19	0.17	Age: 46-64	China	Billed at cost
0.16	0.20	0.18	Age: 65 or over	China	Billed at cost
0.16	0.18	0.17	Living with a child: Yes	China	Billed at cost
0.15	0.17	0.16	Living With a child: No	China	Billed at cost
0.16	0.19	0.18	Living with an elderly individual: Yes	China	Billed at cost
0.14	0.17	0.15	Living with an elderly individual: No	China	Billed at cost
0.14	0.17	0.16	Duration of stay: 0–1 year	China	Billed at cost
0.14	0.18	0.16	Duration of stay: 1–5 years	China	Billed at cost
0.14	0.18	0.16	Duration of stay: 5–10 years	China	Billed at cost
0.16	0.20	0.18	Duration of stay: Longer than 10 years	China	Billed at cost
0.13	0.17	0.15	Visa: Short-term	China	Billed at cost
0.16	0.20	0.18	Visa: Highly skilled	China	Billed at cost
0.17	0.21	0.19	Visa: Working	China	Billed at cost
0.17	0.21	0.19	Visa: Permanent	China	Billed at cost
0.09	0.13	0.11	Visa: Illegal	China	Billed at cost
0.31	0.37	0.34	Occupation: Healthcare	Taiwan	Billed at cost
0.19	0.24	0.21	Occupation: Education/childcare	Taiwan	Billed at cost
0.11	0.15	0.13	Occupation: Other employed	Taiwan	Billed at cost
0.10	0.14	0.12	Occupation: Self-employed	Taiwan	Billed at cost
0.06	0.09	0.07	Occupation: Not employed	Taiwan	Billed at cost
0.13	0.16	0.15	Age: 17-30	Taiwan	Billed at cost
0.15	0.19	0.17	Age: 31-45	Taiwan	Billed at cost
0.17	0.20	0.18	Age: 46-64	Taiwan	Billed at cost
0.18	0.22	0.20	Age: 65 or over	Taiwan	Billed at cost
0.17	0.20	0.19	Living with a child: Yes	Taiwan	Billed at cost
0.15	0.18	0.17	Living With a child: No	Taiwan	Billed at cost
0.17	0.20	0.19	Living with an elderly individual: Yes	Taiwan	Billed at cost
0.15	0.18	0.17	Living with an elderly individual: No	Taiwan	Billed at cost
0.15	0.19	0.17	Duration of stay: 0–1 year	Taiwan	Billed at cost
0.16	0.19	0.17	Duration of stay: 1–5 years	Taiwan	Billed at cost
0.16	0.20	0.18	Duration of stay: 5–10 years	Taiwan	Billed at cost
0.16	0.20	0.18	Duration of stay: Longer than 10 years	Taiwan	Billed at cost
0.15	0.18	0.17	Visa: Short-term	Taiwan	Billed at cost
0.17	0.22	0.20	Visa: Highly skilled	Taiwan	Billed at cost
0.18	0.23	0.20	Visa: Working	Taiwan	Billed at cost
0.18	0.22	0.20	Visa: Permanent	Taiwan	Billed at cost
0.10	0.13	0.12	Visa: Illegal	Taiwan	Billed at cost
0.29	0.34	0.32	Occupation: Healthcare	South Korea	Billed at cost
0.18	0.22	0.20	Occupation: Education/childcare	South Korea	Billed at cost
0.09	0.13	0.11	Occupation: Other employed	South Korea	Billed at cost
0.09	0.12	0.10	Occupation: Self-employed	South Korea	Billed at cost
0.06	0.09	0.07	Occupation: Not employed	South Korea	Billed at cost
0.14	0.17	0.16	Age: 17-30	South Korea	Billed at cost
0.13	0.17	0.15	Age: 31-45	South Korea	Billed at cost
0.14	0.18	0.16	Age: 46-64	South Korea	Billed at cost
0.16	0.19	0.18	Age: 65 or over	South Korea	Billed at cost
0.15	0.18	0.16	Living with a child: Yes	South Korea	Billed at cost
0.14	0.17	0.16	Living With a child: No	South Korea	Billed at cost
0.16	0.19	0.17	Living with an elderly individual: Yes	South Korea	Billed at cost
0.14	0.16	0.15	Living with an elderly individual: No	South Korea	Billed at cost
0.14	0.18	0.16	Duration of stay: 0–1 year	South Korea	Billed at cost
0.14	0.17	0.15	Duration of stay: 1–5 years	South Korea	Billed at cost
0.15	0.18	0.16	Duration of stay: 5–10 years	South Korea	Billed at cost
0.15	0.18	0.16	Duration of stay: Longer than 10 years	South Korea	Billed at cost
0.13	0.17	0.15	Visa: Short-term	South Korea	Billed at cost
0.16	0.20	0.18	Visa: Highly skilled	South Korea	Billed at cost
0.16	0.21	0.19	Visa: Working	South Korea	Billed at cost
0.15	0.20	0.17	Visa: Permanent	South Korea	Billed at cost
0.09	0.13	0.11	Visa: Illegal	South Korea	Billed at cost
0.36	0.41	0.39	Occupation: Healthcare	EU	Billed at cost
0.20	0.25	0.23	Occupation: Education/childcare	EU	Billed at cost
0.13	0.16	0.15	Occupation: Other employed	EU	Billed at cost
0.11	0.14	0.12	Occupation: Self-employed	EU	Billed at cost
0.07	0.10	0.09	Occupation: Not employed	EU	Billed at cost
0.16	0.20	0.18	Age: 17-30	EU	Billed at cost
0.16	0.20	0.18	Age: 31-45	EU	Billed at cost
0.18	0.22	0.20	Age: 46-64	EU	Billed at cost
0.20	0.24	0.22	Age: 65 or over	EU	Billed at cost
0.20	0.23	0.21	Living with a child: Yes	EU	Billed at cost
0.17	0.19	0.18	Living With a child: No	EU	Billed at cost
0.20	0.22	0.21	Living with an elderly individual: Yes	EU	Billed at cost
0.17	0.20	0.18	Living with an elderly individual: No	EU	Billed at cost
0.17	0.21	0.19	Duration of stay: 0–1 year	EU	Billed at cost
0.17	0.20	0.18	Duration of stay: 1–5 years	EU	Billed at cost
0.17	0.21	0.19	Duration of stay: 5–10 years	EU	Billed at cost
0.19	0.23	0.21	Duration of stay: Longer than 10 years	EU	Billed at cost
0.17	0.21	0.19	Visa: Short-term	EU	Billed at cost
0.20	0.24	0.22	Visa: Highly skilled	EU	Billed at cost
0.20	0.24	0.22	Visa: Working	EU	Billed at cost
0.19	0.24	0.22	Visa: Permanent	EU	Billed at cost
0.12	0.16	0.14	Visa: Illegal	EU	Billed at cost
0.33	0.38	0.36	Occupation: Healthcare	US	Government-subsidized
0.20	0.24	0.22	Occupation: Education/childcare	US	Government-subsidized
0.11	0.14	0.13	Occupation: Other employed	US	Government-subsidized
0.11	0.14	0.12	Occupation: Self-employed	US	Government-subsidized
0.06	0.09	0.08	Occupation: Not employed	US	Government-subsidized
0.14	0.18	0.16	Age: 17-30	US	Government-subsidized
0.16	0.20	0.18	Age: 31-45	US	Government-subsidized
0.15	0.19	0.17	Age: 46-64	US	Government-subsidized
0.19	0.23	0.21	Age: 65 or over	US	Government-subsidized
0.17	0.20	0.19	Living with a child: Yes	US	Government-subsidized
0.16	0.19	0.18	Living With a child: No	US	Government-subsidized
0.19	0.22	0.20	Living with an elderly individual: Yes	US	Government-subsidized
0.15	0.18	0.16	Living with an elderly individual: No	US	Government-subsidized
0.15	0.19	0.17	Duration of stay: 0–1 year	US	Government-subsidized
0.16	0.19	0.18	Duration of stay: 1–5 years	US	Government-subsidized
0.17	0.20	0.19	Duration of stay: 5–10 years	US	Government-subsidized
0.17	0.21	0.19	Duration of stay: Longer than 10 years	US	Government-subsidized
0.15	0.19	0.17	Visa: Short-term	US	Government-subsidized
0.18	0.22	0.20	Visa: Highly skilled	US	Government-subsidized
0.19	0.23	0.21	Visa: Working	US	Government-subsidized
0.19	0.23	0.21	Visa: Permanent	US	Government-subsidized
0.10	0.14	0.12	Visa: Illegal	US	Government-subsidized
0.29	0.34	0.32	Occupation: Healthcare	China	Government-subsidized
0.17	0.22	0.20	Occupation: Education/childcare	China	Government-subsidized
0.10	0.14	0.12	Occupation: Other employed	China	Government-subsidized
0.09	0.12	0.10	Occupation: Self-employed	China	Government-subsidized
0.06	0.08	0.07	Occupation: Not employed	China	Government-subsidized
0.12	0.15	0.14	Age: 17-30	China	Government-subsidized
0.14	0.17	0.16	Age: 31-45	China	Government-subsidized
0.15	0.18	0.16	Age: 46-64	China	Government-subsidized
0.17	0.21	0.19	Age: 65 or over	China	Government-subsidized
0.16	0.18	0.17	Living with a child: Yes	China	Government-subsidized
0.14	0.17	0.15	Living With a child: No	China	Government-subsidized
0.16	0.19	0.18	Living with an elderly individual: Yes	China	Government-subsidized
0.13	0.16	0.15	Living with an elderly individual: No	China	Government-subsidized
0.14	0.18	0.16	Duration of stay: 0–1 year	China	Government-subsidized
0.13	0.17	0.15	Duration of stay: 1–5 years	China	Government-subsidized
0.15	0.18	0.17	Duration of stay: 5–10 years	China	Government-subsidized
0.15	0.18	0.17	Duration of stay: Longer than 10 years	China	Government-subsidized
0.12	0.16	0.14	Visa: Short-term	China	Government-subsidized
0.17	0.22	0.20	Visa: Highly skilled	China	Government-subsidized
0.17	0.21	0.19	Visa: Working	China	Government-subsidized
0.16	0.20	0.18	Visa: Permanent	China	Government-subsidized
0.09	0.12	0.11	Visa: Illegal	China	Government-subsidized
0.32	0.37	0.35	Occupation: Healthcare	Taiwan	Government-subsidized
0.19	0.24	0.22	Occupation: Education/childcare	Taiwan	Government-subsidized
0.10	0.14	0.12	Occupation: Other employed	Taiwan	Government-subsidized
0.10	0.13	0.11	Occupation: Self-employed	Taiwan	Government-subsidized
0.06	0.09	0.08	Occupation: Not employed	Taiwan	Government-subsidized
0.13	0.17	0.15	Age: 17-30	Taiwan	Government-subsidized
0.15	0.19	0.17	Age: 31-45	Taiwan	Government-subsidized
0.17	0.20	0.18	Age: 46-64	Taiwan	Government-subsidized
0.17	0.21	0.19	Age: 65 or over	Taiwan	Government-subsidized
0.17	0.20	0.18	Living with a child: Yes	Taiwan	Government-subsidized
0.15	0.18	0.17	Living With a child: No	Taiwan	Government-subsidized
0.17	0.20	0.18	Living with an elderly individual: Yes	Taiwan	Government-subsidized
0.15	0.18	0.17	Living with an elderly individual: No	Taiwan	Government-subsidized
0.15	0.19	0.17	Duration of stay: 0–1 year	Taiwan	Government-subsidized
0.15	0.19	0.17	Duration of stay: 1–5 years	Taiwan	Government-subsidized
0.16	0.20	0.18	Duration of stay: 5–10 years	Taiwan	Government-subsidized
0.16	0.20	0.18	Duration of stay: Longer than 10 years	Taiwan	Government-subsidized
0.14	0.18	0.16	Visa: Short-term	Taiwan	Government-subsidized
0.18	0.23	0.21	Visa: Highly skilled	Taiwan	Government-subsidized
0.18	0.22	0.20	Visa: Working	Taiwan	Government-subsidized
0.18	0.22	0.20	Visa: Permanent	Taiwan	Government-subsidized
0.09	0.12	0.11	Visa: Illegal	Taiwan	Government-subsidized
0.30	0.35	0.32	Occupation: Healthcare	South Korea	Government-subsidized
0.17	0.21	0.19	Occupation: Education/childcare	South Korea	Government-subsidized
0.09	0.13	0.11	Occupation: Other employed	South Korea	Government-subsidized
0.09	0.12	0.11	Occupation: Self-employed	South Korea	Government-subsidized
0.05	0.08	0.07	Occupation: Not employed	South Korea	Government-subsidized
0.12	0.15	0.14	Age: 17-30	South Korea	Government-subsidized
0.13	0.17	0.15	Age: 31-45	South Korea	Government-subsidized
0.15	0.18	0.16	Age: 46-64	South Korea	Government-subsidized
0.17	0.21	0.19	Age: 65 or over	South Korea	Government-subsidized
0.15	0.18	0.17	Living with a child: Yes	South Korea	Government-subsidized
0.14	0.17	0.15	Living With a child: No	South Korea	Government-subsidized
0.16	0.18	0.17	Living with an elderly individual: Yes	South Korea	Government-subsidized
0.14	0.16	0.15	Living with an elderly individual: No	South Korea	Government-subsidized
0.14	0.17	0.16	Duration of stay: 0–1 year	South Korea	Government-subsidized
0.14	0.18	0.16	Duration of stay: 1–5 years	South Korea	Government-subsidized
0.14	0.18	0.16	Duration of stay: 5–10 years	South Korea	Government-subsidized
0.15	0.19	0.17	Duration of stay: Longer than 10 years	South Korea	Government-subsidized
0.13	0.16	0.14	Visa: Short-term	South Korea	Government-subsidized
0.16	0.20	0.18	Visa: Highly skilled	South Korea	Government-subsidized
0.16	0.21	0.18	Visa: Working	South Korea	Government-subsidized
0.16	0.21	0.19	Visa: Permanent	South Korea	Government-subsidized
0.09	0.12	0.11	Visa: Illegal	South Korea	Government-subsidized
0.34	0.39	0.36	Occupation: Healthcare	EU	Government-subsidized
0.22	0.26	0.24	Occupation: Education/childcare	EU	Government-subsidized
0.12	0.15	0.13	Occupation: Other employed	EU	Government-subsidized
0.10	0.13	0.12	Occupation: Self-employed	EU	Government-subsidized
0.06	0.09	0.08	Occupation: Not employed	EU	Government-subsidized
0.16	0.19	0.17	Age: 17-30	EU	Government-subsidized
0.15	0.19	0.17	Age: 31-45	EU	Government-subsidized
0.17	0.20	0.19	Age: 46-64	EU	Government-subsidized
0.19	0.23	0.21	Age: 65 or over	EU	Government-subsidized
0.18	0.21	0.19	Living with a child: Yes	EU	Government-subsidized
0.16	0.19	0.18	Living With a child: No	EU	Government-subsidized
0.18	0.21	0.19	Living with an elderly individual: Yes	EU	Government-subsidized
0.17	0.19	0.18	Living with an elderly individual: No	EU	Government-subsidized
0.16	0.20	0.18	Duration of stay: 0–1 year	EU	Government-subsidized
0.16	0.20	0.18	Duration of stay: 1–5 years	EU	Government-subsidized
0.18	0.22	0.20	Duration of stay: 5–10 years	EU	Government-subsidized
0.16	0.20	0.18	Duration of stay: Longer than 10 years	EU	Government-subsidized
0.14	0.18	0.16	Visa: Short-term	EU	Government-subsidized
0.18	0.23	0.21	Visa: Highly skilled	EU	Government-subsidized
0.20	0.24	0.22	Visa: Working	EU	Government-subsidized
0.19	0.24	0.22	Visa: Permanent	EU	Government-subsidized
0.10	0.13	0.12	Visa: Illegal	EU	Government-subsidized
					

**Table A6:** Demographic summary of the national census of 2020.

			
Population	Total	Men	Women
	126,146,099	61,349,581	64,796,518
	100.0%	48.6%	51.4%
Age	Median	47.1	50
	Mean	46.0	49.2
Labor participation and marital status: 15–64 years old		Men	Women
Population: 15–64 years old	*a*	36,753,516	36,169,248
Labor market participants	*b*	27,609,467	23,343,225
Population at work	*c*	26,396,754	22,521,997
Unknown	*d*	4,950,783	4,129,413
Labor participation rate: 15–64 years old	*b*/(*b* − *d*)	86.8%	72.9%
Marital status	Unmarried	14,827,517	11,790,437
	Married	18,411,345	20,211,842
	Bereaved/divorced	1,516,734	2,892,487
Education		Men	Women
Population: 24–64 years old	*e*	33,873,487	33,431,837
Highest degree: Elementary	*f*	11,428	12,435
Highest degree: Junior high school	*g*	1,769,706	1,182,121
Highest degree: High school	*h*	11,378,052	11,367,250
Highest degree: 2-year college	*i*	3,238,808	7,922,954
Highest degree: 4-year college	*j*	9,480,016	6,380,243
Highest degree: Graduate school	*k*	1,364,980	458,009
In school	*l*	1,231,413	1,075,824
Tertiary educated	(*i* + *j* + *k*)/*e*	41.6%	44.2%

*Source*: 2020 Population Census, administered by the Ministry of Internal Affairs and Communications of the Government of Japan (https://www.e-stat.go.jp/en/stat-search/files?page=1&toukei=00200521&tstat=000001136464. Last accessed on June 30, 2022).

**Table A7:** Distribution of household income from the National Livelihood Survey

Income level	Number	Share
Total	10,000	100.00%
Less than JPY0.5 million	120	1.20%
JPY0.5–1 million	519	5.19%
JPY1–1.5 million	631	6.31%
JPY1.5–2 million	632	6.32%
JPY2–2.5 million	689	6.89%
JPY2.5–3 million	666	6.66%
JPY3–3.5 million	711	7.11%
JPY3.5–4 million	574	5.74%
JPY4–4.5 million	555	5.55%
JPY4.5–5 million	491	4.91%
JPY5–5.5 million	488	4.88%
JPY5.5–6 million	380	3.80%
JPY6–6.5 million	463	4.63%
JPY6.5–7 million	344	3.44%
JPY7–7.5 million	329	3.29%
JPY7.5–8 million	288	2.88%
JPY8–8.5 million	260	2.60%
JPY8.5–9 million	232	2.32%
JPY9–9.5 million	216	2.16%
JPY9.5–10 million	185	1.85%
More than JPY10 million	1,225	12.25%

*Source*: National Livelihood Survey 2019, administered by the Ministry of Health, Labour and Welfare of the Government of Japan (https://www.e-stat.go.jp/stat-search/file-download?statInfId=000031957851&fileKind=1. Last accessed on July 5, 2021).

## Data Availability

Data will be made available on request.

## Appendix

Table A1 presents the point estimates for the ranking of hypothetical vaccine recipients, $\tau^{\mathrm{order}}\left(A_{j}^{i,r}\right)$, as characterized by equation (2), the associated 95% clustering robust confidence interval without multiple testing adjustment, the hypothetical recipients’ attributes, and their citizenship status, as shown in Fig. 1. As indicated in the caption for Fig. 1, a smaller point estimate for the ranking indicates a more highly prioritized candidate.

Table A2 presents the estimated probability that foreigners are prioritized over Japanese citizens on average, $\tau^{\mathrm{FoverJ}}\left(A_{F,j}^{i,r},A_{J,j^{\prime }}^{i,r}\right)$, as characterized by equation (4), the associated 95% confidence intervals, the hypothetical vaccine recipients’ attributes, and their citizenship status, as shown in Fig. 2. A greater point estimate indicates a higher estimated probability of being prioritized over Japanese citizens on average.

Table A3 presents the estimated probability that foreigners are prioritized over Japanese citizens when foreign and Japanese candidates’ occupation are the same, $\tau^{\mathrm{FoverJ}}\left(A_{F,j}^{i,r},A_{J,j^{\prime }}^{i,r}\right)$, as characterized by equation (4), the associated 95% confidence intervals, the hypothetical vaccine recipients’ attributes, and their citizenship status, as shown in Fig. 3. A greater point estimate indicates a higher estimated probability of being prioritized over Japanese citizens on average.

Table A4 presents the difference in the estimated probability of prioritizing foreigners over Japanese citizens on average by respondent gender, $\underset{i_{f},i_{m}}{\Delta }\tau^{\mathrm{FoverJ}}\left(A_{F,j}^{i,r},A_{J,j^{\prime }}^{i,r}\right)$ from equation (9), the 95% confidence intervals, the hypothetical vaccine recipients’ attributes, and their citizenship status, as shown in Fig. 4. Estimates indicate the estimated probability of female respondents prioritizing foreigners $\tau^{\mathrm{FoverJ}}\left(A_{F,j}^{i_{f},r},A_{J,j^{\prime }}^{i_{f},r}\right)$, from which the estimated probability of male respondents prioritizing foreigners $\tau^{\mathrm{FoverJ}}\left(A_{F,j}^{i_{m},r},A_{J,j^{\prime }}^{i_{m},r}\right)$ is subtracted. A larger estimate indicates that female respondents are on average likely to prioritize foreign recipients over Japanese recipients to a greater extent than male respondents are.

Table A5 presents the estimated probability that foreign recipients are prioritized over Japanese recipients on average, the associated the 95% confidence intervals, the hypothetical vaccine recipients’ attributes, and their citizenship status under two vaccine payment scenarios randomly shown to respondents, billed at cost and government subsidized, as shown in Fig. 5.

Table A6 presents a demographic summary of the national census of 2020 administered by the Ministry of Internal Affairs and Communications of the Government of Japan, for comparison with our sample. As Table 5 shows, our sample respondents are slightly more educated.

Finally, Table A7 shows the income distribution of 10,000 households surveyed as part of the National Livelihood Survey administered by the Ministry of Health, Labour and Welfare of the Government of Japan^3^ for comparison with our sample presented in Table 6.

## Footnotes

1 A press release from the Ministry of Defense can be seen at https://www.mod.go.jp/en/index.html#security-cooperation. Accessed March 4, 2022.

2 Detailed information about Rakuten Insight’s respondent pool is available from its website. https://insight.rakuten.co.jp/download/PanelProfile_EN.pdf and https://insight.rakuten.co.jp/download/PanelCharacteristicSurveyEN.pdf.
